# Fin Spine Bone Resorption in Atlantic Bluefin Tuna, *Thunnus thynnus*, and Comparison between Wild and Captive-Reared Specimens

**DOI:** 10.1371/journal.pone.0121924

**Published:** 2015-03-09

**Authors:** Nicoletta Santamaria, Giambattista Bello, Chrysovalentinos Pousis, Robert Vassallo-Agius, Fernando de la Gándara, Aldo Corriero

**Affiliations:** 1 Department of Emergency and Organ Transplantation, Section of Veterinary Medicine and Animal Production, University of Bari Aldo Moro, Valenzano (BA), Italy; 2 Malta Aquaculture Research Centre, Marsaxlokk, Malta; 3 Centro Oceanográfico de Murcia, Instituto Español de Oceanografía, Puerto de Mazarrón, Spain; Rensselaer Polytechnic Institute, UNITED STATES

## Abstract

Bone resorption in the first spine of the first dorsal fin of Atlantic bluefin tuna (ABFT) has long been considered for age estimation studies. In the present paper spine bone resorption was assessed in wild (aged 1 to 13 years) and captive-reared (aged 2 to 11 years) ABFT sampled from the Mediterranean Sea. Total surface (*TS*), solid surface (*SS*) and reabsorbed surface (*RS*) were measured in spine transverse sections in order to obtain proportions of *SS* and *RS*. The spine section surface was found to be isometrically correlated to the fish fork length by a power equation. The fraction of solid spine bone progressively decreased according to a logarithmic equation correlating *SS/TS* to both fish size and age. The values ranged from 57% in the smallest examined individuals to 37% in the largest specimens. This phenomenon was further enhanced in captive-reared ABFT where *SS/TS* was 22% in the largest measured specimen. The difference between the fraction of *SS* of wild and captive-reared ABFT was highly significant. In each year class from 1- to 7-year-old wild specimens, the fraction of spine reabsorbed surface was significantly higher in specimens collected from March to May than in those sampled during the rest of the year. In 4-year-old fish the normal *SS* increase during the summer did not occur, possibly coinciding with their first sexual maturity. According to the correlations between *SS/TS* and age, the rate of spine bone resorption was significantly higher, even almost double, in captive-reared specimens. This could be attributed to the wider context of systemic dysfunctions occurring in reared ABFT, and may be related to a number of factors, including nutritional deficiencies, alteration of endocrine profile, cortisol-induced stress, and loss of spine functions during locomotion in rearing conditions.

## Introduction

The Atlantic bluefin tuna (ABFT), *Thunnus thynnus* (Linnaeus, 1758) (Osteichthyes: Scombridae), is one of the fastest, largest and long-lived teleost fish. It can perform trans-Atlantic migrations and swim at 90 km per hour [1–5]. The ABFT, like other tuna species, have very peculiar physiological characteristics, such as the ability to elevate the temperature of their locomotory muscles, viscera, brain and eye tissues above that of the ambient water (regional endothermy) [6–10].

The ABFT has historically been an important economic resource in the Atlantic Ocean as well as in the Mediterranean Sea, where different fishing strategies have been developed. At present, the bluefin tuna is one of the most valuable fish due to its high prices on the Japanese market, where its flesh represents the basis of highly prized delicacies such as sushi and sashimi [11]. In the last 15 years, a capture-based aquaculture industry which concentrates on the capture of juvenile (tuna farming) or adult (tuna fattening) individuals and their rearing for a few years or a few months, respectively, before harvesting has developed in the Mediterranean Sea [11]. Due to this industry, several European research institutions, in collaboration with the tuna farming and fattening industry, have participated in attempts to domesticate the ABFT since the early 2000’s [12–24].

When reared in captivity, many fish show a variety of pathologies or dysfunctions that may be attributable, among other causes, to altered social relationships [25–27], inadequate environmental factors [28–29] or nutritional deficiencies [13, 30–32]. In the case of adult ABFT, an impairment of the reproductive axis has been documented when reared in captivity [12–14, 22, 23, 33]. Further, an increase of melanomacrophagic centers, apoptosis and tumor necrosis factor gene expression have been reported in the liver of juvenile ABFT reared in waters potentially exposed to environmental pollutants [34, 35].

A variety of methods can be used for the age estimation of fish species and they usually include the reading of hard parts, such as otoliths, scales, spines and vertebrae. These methods are based on the number of marks when examined transversally, usually called annuli, which are interpreted as periodic events [36, 37]. The ABFT is provided with median (dorsal and anal) and paired (pectoral and pelvic) fins. Of the two dorsal fins, the cranial one or first dorsal fin is supported by 12–15 spiny rays (spines), the caudal one or second dorsal fin is made of a spine followed by 11–13 soft rays (rays) [38]. The first spine of the first dorsal fin is the most suitable for age determination studies because its transverse sections display well-defined growth marks and it can be easily collected [24, 37, 39–42]. The presence of the growth marks is due to the progressive apposition of bone tissue on the external side of the spine, which becomes apparent as an ordered series of alternate opaque and translucent rings, corresponding to a faster spring-summer and a slower autumn-winter growth, respectively [37, 39–41]. The optical differences between translucent and opaque rings are related to different calcium concentrations, with higher concentrations in the translucent ones [43]. In the ABFT, concomitantly with bone apposition on the external side, a physiological progressive resorption of bone tissue from the inner part of the spine (the so called core or nucleus) occurs [37, 39–41]. A recent study [42] thoroughly reviewed the literature on the use of the first dorsal spine to age ABFT and discussed the problem of bone resorption. It also emphasized the absence of any study regarding the quantification of obscured annuli due to bone resorption. Apart from the need to quantify the spine bone resorption process in order to obtain more accurate age estimates, the understanding of such a process should be linked to the physiology of this fish in relation to its feeding, reproduction, migrating and growing characteristics.

The present study was prompted by observations from a comparative study of age and growth of wild and captive-reared ABFT, when it was well noted that the spine bone resorption process was more marked in specimens reared in captivity. Following this basis, this study aims to: 1) model the bone resorption progress in the first spine of the first dorsal fin of wild ABFT during growth; 2) describe seasonal differences in the bone resorption/deposition process and 3) compare the spine resorption process of wild and captive-reared Atlantic bluefin tuna.

## Materials and Methods

**Fig 1:**
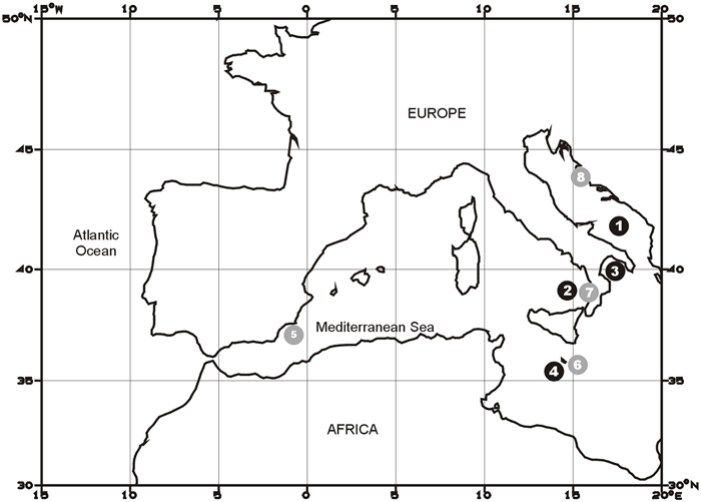
Approximate geographical location of sampling areas for wild and captive-reared Atlantic bluefin tuna. Black and grey circles indicate sampling sites for wild and captive-reared specimens, respectively. 1, South Adriatic Sea; 2, South Tyrrhenian Sea; 3, North Ionian Sea (Gulf of Taranto); 4, Ionian Sea around Malta; 5, Puerto de Mazarrón and Cartagena, Spain; 6; Malta; 7, Vibo Marina, Italy; 8, Drvenik and Uglyan Island, Croatia.

Wild and captive-reared ABFT (n = 186 and 242, respectively) were sampled over the eight-year period 2003–2010 in several sites of the Mediterranean Sea (Fig. 1). Wild fish were caught by commercial long-liners and purse seiners whereas captive-reared specimens were sampled in the framework of three research projects aimed at ABFT domestication (EU project REPRODOTT, EU project SELFDOTT and Italian project ALLOTUNA funded by the regional government of the Apulia region).

**Fig 2:**
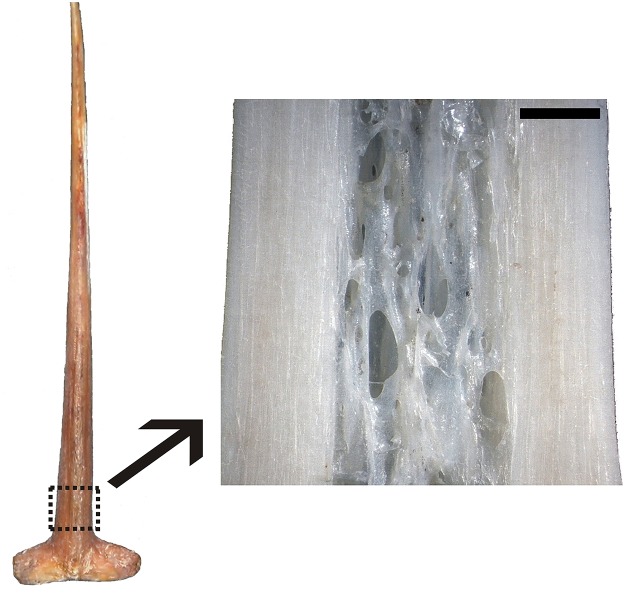
Rostral aspect of the first ray of the first dorsal fin from a captive-reared Atlantic bluefin tuna with 166 cm *FL* and 7 years estimated age. The dashed rectangle indicates the region of the spine above the condyle from which a frontal section was cut (micrograph on the right). The spine structure consists of an external compact bone and an internal woven zone that increases with growth. The spine length is 17 cm; the magnification bar of the spine section represents 2 cm.

From each fish the fork length, *FL*, was measured to the nearest cm and the first spine of the first dorsal fin was removed (Fig. 2). A low speed diamond saw (Buehler, Isomet) was used to obtain a cross-section, about 0.7 mm thick. The cut was carried out at a distance of half the maximum spine diameter from the condyle base, as commonly performed in age determination studies [42], and were mounted with Eukitt Mounting Medium (Electron Microscopy Sciences, Hatfield, PA, U.S.A.) on glass slides.

**Fig 3:**
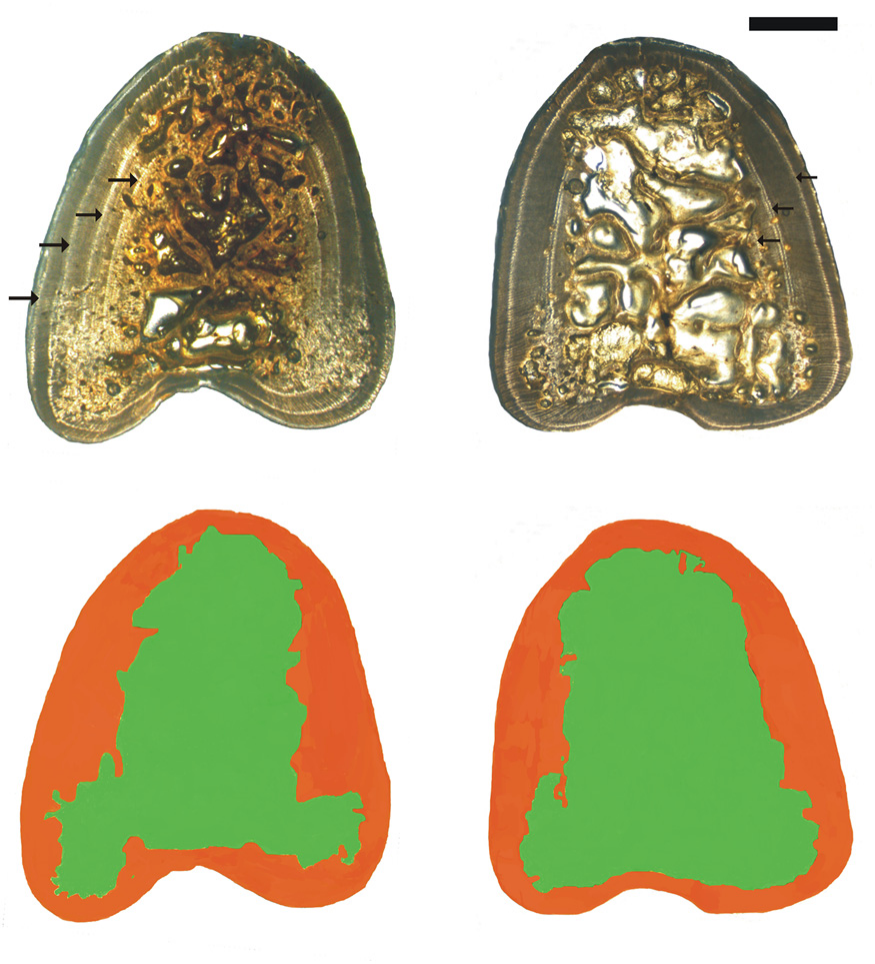
Cross section of the first ray of the first dorsal fin from two 6-year-old Atlantic bluefin tuna specimens. a) Wild specimen, *FL* = 154 cm. Arrows indicate the four visible annuli, 3^rd^ to 6^th^; the first two annuli were reabsorbed. b) Captive-reared specimen, *FL* = 156 cm. Arrows indicate the three visible annuli (4^th^ to 6^th^); the first three annuli were reabsorbed. The sections were cut above the spine condyle. c) and d) schematic view of photographs in a) and b), respectively, with unabsorbed bone areas highlighted in green and partially or totally reabsorbed areas in orange. Note the larger extension of the green area in the captive-reared specimen with respect to the wild one, which indicates a higher degree of bone resorption.

An age was assigned to each fish by counting the narrow translucent and wider opaque zones that represent periods of slow and fast growth, respectively [36, 37, 39–41]. Hence, a translucent zone and the associated opaque zone together were assumed to represent an annual growth band. Since it is known that the core of the spine is progressively reabsorbed and the first rings begin to disappear at age 3, the mean diameters of the first rings of younger specimens were used to ascertain the age of the first visible ring of older specimens [41, 42] (Fig. 3). Due to the fact that the mean annulus diameter estimates for wild and captive ABFT differed slightly from each other, two different sets of mean values were used.

The following measurements in cm^2^ were taken on each spine section: *TS*: total surface; *SS*: solid part surface (non-reabsorbed bone tissue with dark appearance); *RS*: reabsorbed part surface (partially or totally reabsorbed bone tissue with grey to white appearance) (Fig. 3). Measurements were performed on spine section images, using an interactive function (i.e. measurements of operator-selected surfaces by a specific image analysis software function), by means of image analysis software Quantiment 500 W (Leica, Wetzlar, Germany), photographed with a 6.4 objective with a digital camera DC 300 (Leica, Wetzlar, Germany) connected to a binocular lens microscope Wild M3C (Leitz, Heerbrugg, Switzerland).

The correlation between the total surface of the spine section (*TS*) and fish fork length (*FL*) was examined and differences between male and female correlation equations were tested.

The degree of bone resorption for each spine was calculated as 1 less the ratio between solid part and total surface, (1—*SS/TS*). Since teleost bone resorption is a progressive process [44], the correlation of *SS/TS* to *FL* and that of *SS/TS* to age were examined in order to describe the trend of the spine bone resorption during growth.

With reference to age, since it is known that ABFT spawn in June-July [45–47], each wild caught individual was assigned an age accurate to a quarter of a year by taking into account the month of capture, in addition to the spine section age class estimation. Hence age class 1 specimens sampled in the June-August quarter were aged 1.0, those collected from September to November 1.25, those from December to February 1.5, those from March to May 1.75; and so on for the following age classes. This subdivision allowed the examination of seasonal differences in the ratio *SS/TS* within each age class. No seasonal sub-division was done for the captive reared tunas samples as they were all sacrificed and their spine was collected during June or July.

In order to understand whether the spine erosion progress through age is statistically significant, the *SS/TS* values of the different age classes were compared by ANOVA.

The statistical probability significance was established at the *P* ≤ 0.05 level.

## Results

### Spine macroscopic morphology and structure

The first spine of the first dorsal fin is an elongated rod articulated to the radial bone by means of a condyle and progressively tapered at its distal end (Fig. 2). A membrane connects the concavity of the first spine to the cranial margin, concave itself, of the following one.

The spine cross section has a cranial rounded apex and a caudal concave base as seen in Fig. 3. In cross sections, the external zone is characterized by solid bone tissue made of alternating translucent and opaque bands while the inner woven bone zone shows irregular apparent cavities (the spines were not fixed and soft tissue had degenerated) among anastomosing bone trabeculae (Fig. 3). The spine frontal section appears as two external layered, solid zones sandwiching an inner woven zone (Fig. 2). Spine inner bone erosion was evident in all 1-year-old wild ABFT, i.e. the youngest available specimens, which shows that it is an early phenomenon. The integration of cross, frontal and sagittal sections indicates that the new bone material is deposed on the whole spine external surface as to progressively envelop it, so that the spine grows both in width and in length.

The overall estimated age of the ABFT sampled in the present study ranged from 1 to 13 years as shown in Table 1.

**Table 1:** Number of examined specimens subdivided per age.

Wild		Captive	
Age (years)	n	Age (years)	n
1.25	5	2	10
1.75	8	3	25
2.25	1	4	41
2.75	34	5	45
3.25	14	6	35
3.75	12	7	44
4.25	18	8	20
4.75	16	9	14
5.25	15	10	7
5.75	7	11	1
6.25	10		
6.75	7		
7.25	3		
7.75	1		
10	5		
11	10		
12	13		
13	7		
**Tot**	**186**	**Tot**	**242**

Age 1.25 year includes wild specimens of age class 1 captured from June to February and so on for the following age classes; age 1.75 year includes wild specimens of age class 1 captured from March to May and so on for the following age classes.

### Wild Atlantic bluefin tuna

According to the annuli count in the spine sections the wild ABFT, their age ranged from 1 to 13 years. There were no specimens of ages 8 or 9 within the sample.

**Fig 4:**
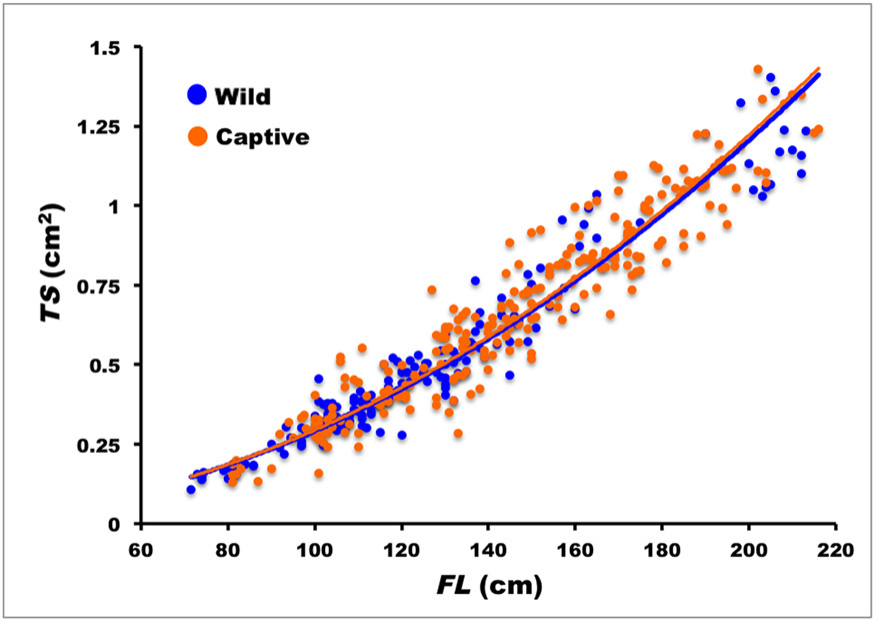
Relationship of total spine surface, *TS*, to fork length, *FL*, for wild (solid line) and captive-reared Atlantic bluefin tuna (broken line).

The surface of the first dorsal spine section increased with size and no significant differences were found between correlations of spine section surface, *TS*, with size, *FL*, for males and females, so all the data were pooled. The relationship between *TS* and *FL* is best described by the power equation *TS* = 3.225×10^-5^ *FL* ^1.980^ (*n* = 186; *s*_*b*_ = 0.033; *r* = 0.976; *P*_*r*_ < 0.0001) (Fig. 4). The slope coefficient (*b* = 1.980) is not significantly different from 2, as expected in a correlation between a surface and a linear size in animals, which shows that the spine section surface grows isometrically with respect to body size.

**Fig 5:**
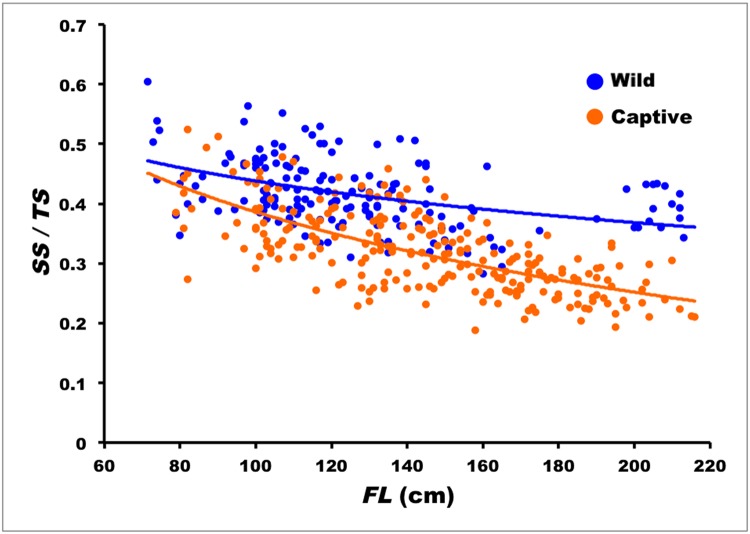
Relationship of solid/total spine surface, *SS/TS*, to fork length, *FL*, for wild (solid line) and captive-reared Atlantic bluefin tuna (broken line).

**Fig 6:**
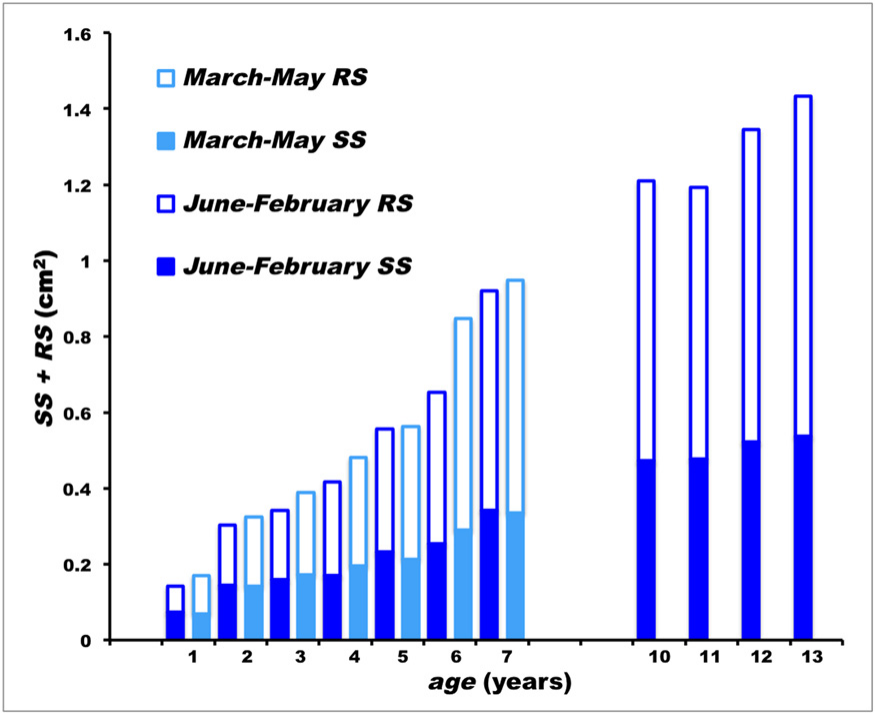
Progress of spine surface during growth in wild Atlantic bluefin tuna. Ages from 1 to 7 years are represented by two columns, one for specimens sampled from June to February, the other one for March to May specimens. *SS*: mean solid fraction of spine section surface; *RS*: mean reabsorbed fraction of spine section surface; *SS + RS*: mean total surface of spine section.

The best model to describe the relationship between *SS/TS* and *FL* was the logarithmic one: *SS/TS* = 0.813–0.0818 ln *FL* (*n* = 186; *s*_*b*_ = 0.0124; *r* = -0.436; *P*_*r*_ < 0.0001) (Fig. 5), indicating that spine erosion, i.e. (*TS* − *SS*), increases with fish size. The average solid fraction in the spine section surface was 52% in the smallest examined individuals (*FL* = 70–75 cm) and decreased to about 37% in the largest specimens (*FL* = 220–240 cm). Moreover according to the logarithmic model, the spine erosion advancement, as measured on the spine section, proceeded at a slower pace as fish size increased (Fig. 6).

**Fig 7:**
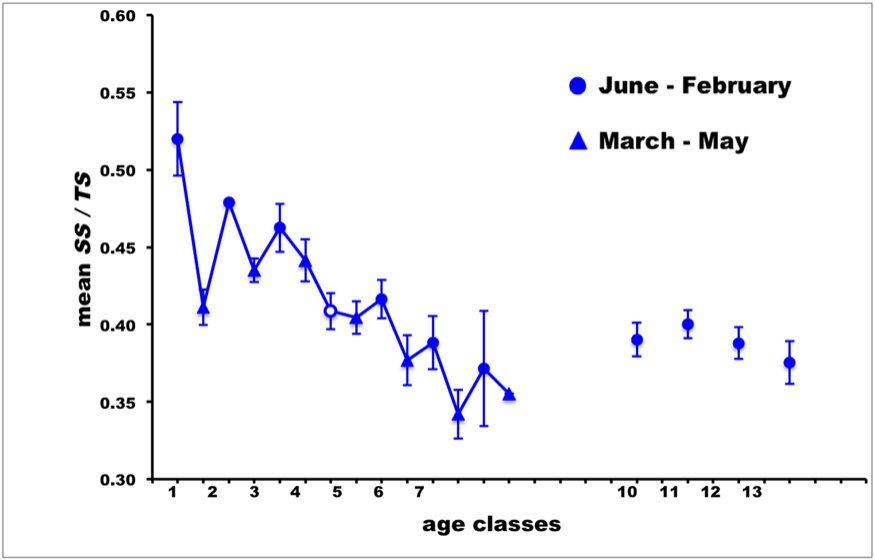
Seasonal trend of mean ratio between spine section solid and total surfaces (*SS/TS*) in wild Atlantic bluefin tuna. The line joining the data points shows the alternating seasonal progress of *SS/TS* means. The hollow circle highlights age 4.25 mean (see text).

Mean *SS/TS* values of all age classes differed significantly from each other (ANOVA: *n* = 185; *F* = 6.682; d.f. = 10/175; *P*_*F*_ < 0.0001). Since the distribution of mean *SS/TS* values for the oldest examined age classes, i.e. 10 to 13, showed little disparity (Fig. 7), ANOVA was also applied independently and no significant difference was detected among the individual year groups (*n* = 35; *F* = 0.685; d.f. = 3/31; *P*_*F*_ = 0.586, n.s.). Hence the erosion phenomenon tended towards equilibrium with new spine material deposition in specimens older than 10 years.

In addition to inter-age class variations, within age class seasonal mean *SS/TS* variations were observed. No significant differences were detected among the *SS/TS* values for the specimens collected in the quarters of year June-August, September-November and December-February, which were pooled together, averaged and compared to the *SS/TS* values for the specimens caught in the March-May quarter of year. The age of these specimens was also averaged, so that the fish of age class 1 collected from June to February were assigned the age 1.25 year and so on for the following year classes. The fish caught from March to May were assigned their actual age approximated to the quarter of year, i.e. 1.75, 2.75 etc. The comparison by ANCOVA applied to the *SS-TS* relationship (with *TS* as covariate) for age classes 1 to 7, of all June-February specimens against all March-May specimens showed that their overall difference was statistically significant (*F*_*s*_ = 5.195; d.f. = 1/148; *P*_*F*_ = 0.024) (data for the age classes 10 to 13 were disregarded because all of them pertain to specimens caught in the June-August quarter of year). The two yearly *SS/TS* means for ages 1.25 to 7.25 and for ages 1.75 to 7.75 are displayed in Fig. 7 along with the only yearly mean for the specimens of age classes 10 to 13.

Figs. 6 and 7 show the marked inner spine resorption until age class 7, with a decrease of mean *SS/TS* in spring and an increase in the following summer through winter, with the exception of age class 4 where no recovery of the solid fraction of spine, as in the years 1 to 3 and 5 to 7, occurred in 4.25-year-old specimens. The mean *SS/TS* value for age 4.25 (0.409) was found to be significantly lower than that for age 3.75 (0.441): *t* = 2.103, d.f. = 28, *P*_*t*_ < 0.05. The decrease of *SS/TS* at age 4.25, in both males and females, represents an exception to the general alternating seasonal pattern (Fig. 7).

The examination of the only pooled June-February *SS/TS* mean values (weighted means), which displayed a regular trend, showed that they are distributed according to a logarithmic equation, namely *SS/TS* = 0.507–0.0527 ln *age* (*n* = 101; *s*_*b*_ = 0.00292; *r* = -0.876; *P*_*r*_ < 0.0001). Despite the possible occurrence of an inflection point in the curve separating the first seven year classes from the last four, there was no actual change in slope between the logarithmic curves for 1–7 and 10–13 age classes (*t*_*slope*_ = 0.813; d.f. = 99; *P*_*t*_ = 0.209, n.s.).

### Captive-reared Atlantic bluefin tuna

In the case of captive-reared ABFT no sex-related differences were found in the relationship between the surface of the 1^st^ dorsal spine section, *TS*, and fork length, *FL*. For the pooled data, this is best described by a power equation: *TS* = 1.900×10^-5^ *FL* ^2.094^ (*n* = 242; *s*_*b*_ = 0.0321; *r* = -0.973; *P*_*r*_ < 0.0001) (Fig. 4).

**Fig 8:**
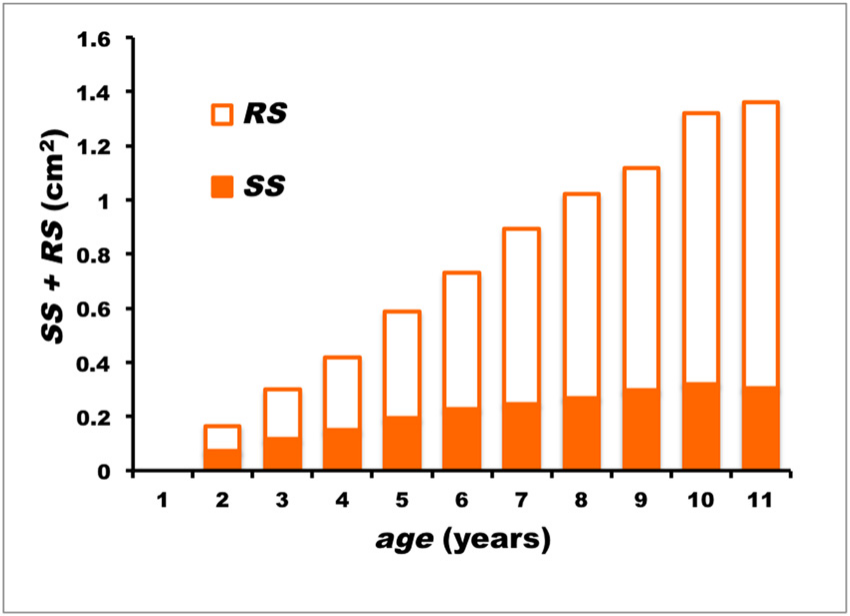
Progress of spine surface during growth in captive-reared Atlantic bluefin tuna. *SS*: mean solid fraction of spine section surface; *RS*: mean reabsorbed fraction of spine section surface; *SS + RS*: mean total surface of spine section.

The logarithmic equation that describes the relationship between *SS/TS* and *FL* in captive-reared ABFT is: *SS/TS* = 1.279–0.194 ln *FL* (*n* = 242; *s*_*b*_ = 0.0135; *r* = -0.680; *P*_*r*_ < 0.0001). As shown in the case of the wild specimens, spine erosion increased with fish size, but at a much faster rate, so that the solid portion of the spine section surface dropped to 22% in the largest specimen (age 11, *FL* = 212 cm) (Fig. 8). The equation line relating *SS/TS* to *FL* in captive-reared ABFT differed significantly from that of wild ABFT (*t*_*slope*_ = 6.098; d.f. = 424; *P* < 0.0001).

**Fig 9:**
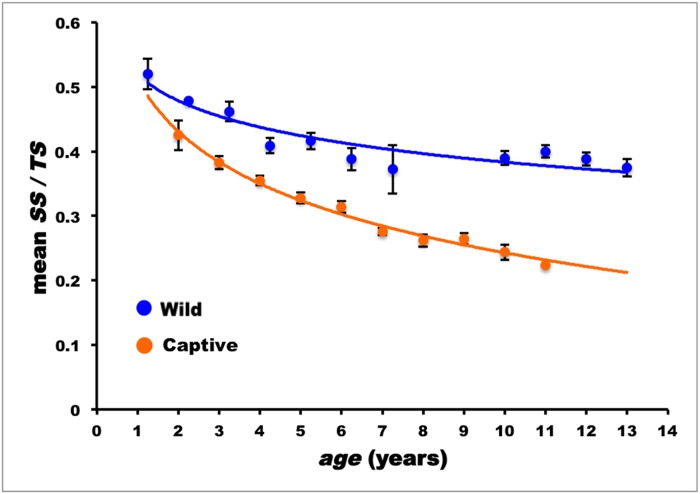
Progress of the mean ratio between spine section solid and total surfaces (*SS/TS*) with age in wild and captive reared Atlantic bluefin tuna; vertical bars: standard error of mean values.

As in wild ABFT, the *SS/TS* decreased with age, so that the overall inter-age classes differences were statistically highly significant (ANOVA: *n* = 242; *F* = 22.579; d.f. = 9/232; *P*_*F*_ < 0.0001). The correlation of weighted *SS/TS* means to age, according to the logarithmic model, *SS/TS* = 0.516–0.118 ln *age* (*n* = 242; *s*_*b*_ = 0.0012; *r* = -0.988; *P*_*r*_ < 0.0001), showed that the rate of *SS/TS* decreased more than double when compared to wild specimens: *b*_*captive*_ = -0.118, *b*_*wild*_ = -0.052 (the first dorsal fin spines of captive ABFT were collected in summer, i.e. when seasonal erosion is at its lowest; cf. results for wild individuals). Fig. 9 shows that the regression lines of the two experimental groups were significantly different (*t*_*slope*_ = 20.866; d.f. = 339; *P* < 0.0001).

## Discussion

The spinous rays of the first dorsal fin of the ABFT, as well as those of other tuna species that inhabit temperate seas such as Atlantic bonito (*Sarda sarda*) [48], bullet tuna (*Auxis rochei*) [49], and albacore (*Thunnus alalunga*) [50] undergo a seasonal bone apposition process that fisheries scientists use for age and growth studies [41]. Along with the process of bone apposition, fin spines are affected by a progressive resorption process that involves the inner part of their structure, a phenomenon that was described more than half a century ago [44, 51]. As regards the ABFT, age determination studies demonstrated that the bone resorption process starts at an early age, so that the first annual ring (the translucent band corresponding to the first year of age) begins to dissolve and its residual fragments are visible when the fish is 3 years old [36, 39–42]. Also the following annual rings progressively disappear with fish growth [41, 42].

To date there is no systematic study on the temporal pattern of bone resorption in any fish species reported. This is the first attempt to model the spine resorption process during the first 13 years of life of wild ABFT, one of the most long-lived fish species. In ABFT, the surface of the section of the first dorsal spine increases with fish size according to an isometric model and a strong correlation between spine size (diameter) and *FL* was also found [42]. This isometric relationship between spine section surface and *FL* substantiated the use of the ratio between the solid surface and the total surface of the spine section (*SS/TS*) as an unbiased measure of bone resorption intensity throughout the whole size range.

According to the data presented in this study, spine bone resorption advances with age so that the fraction of reabsorbed tissue, i.e. (1—*SS/TS*), increases in older and larger specimens, although in wild ones it seems to level off or at least slow down to a great extent in individuals older than 10 years. Incidentally, in these animals the observed decrease in bone resorption is proportionally associated to the decrease of bone apposition. The overall yearly deposition of new bone slightly decreases with growth and less bone is proportionally reabsorbed (data not shown). This is possibly related to the decrease of the growth rate in individuals older than 10 years as described by Luque et al. [42]. Moreover, bone resorption and apposition is a seasonal process. In fact, within each age class the average value of the *SS/TS* ratio is lower in the March-May quarter than in the remaining part of the year. This is likely to result from an unbalanced bone resorption/deposition ratio occurring during winter and the early spring months. This may be correlated to lower water temperatures as well as to the long migrations towards feeding and reproductive grounds that characterize this fish species [2–4, 52, 53]. Vertebral bone resorption during reproductive migration has also been reported in other highly migratory fish like the Atlantic salmon (*Salmo salar*) [54].

Age determination studies for ABFT have reported that the deposition of the translucent growth band (corresponding to slow fish growth) starts in February and lasts until April-May, while the opaque band (corresponding to fast fish growth) is added from late May onwards [37]. This periodicity fits closely with that of the spine bone resorption/apposition process observed in the present paper since bone resorption/deposition is a seasonal process, with resorption prevailing during the winter-spring months and deposition during summer and autumn. This seasonal pattern of the bone resorption/deposition process is dramatically modified between the 3^rd^ and 4^th^ years of life. In fact, the usual summer relative increase of the solid part fraction of the spine detected in cross sections did not occur in fish of the age class 4, resulting in a continuous predominance of bone erosion over bone deposition from the winter of the 3^rd^ year to the end of the 4^th^ year of life. This period of the life cycle of the ABFT corresponds to the first sexual maturity, which in fact, in the Mediterranean, occurs between the 3^rd^ and 5^th^ years of age [40]. It can be hypothesized that the abrupt body changes, usually associated with the onset of sexual maturity, affect the bone resorption/deposition process in some way, probably through the displacement of energy investment towards gamete production [55]. The normal seasonal interchange of winter-spring resorption and summer apposition resumes in the 5^th^ year of life, when 100% of the fish are sexually mature [40].

When comparing captive reared individuals to wild ones, there were significant differences in the spine resorption process. The correlation between *SS/TS* and age is significantly different in captive and wild ABFT, corresponding to an overall bone reabsorbing rate that is almost double for fish reared in captivity. This phenomenon should be dealt with in self-sustaining farming activities, as envisaged for the future [11], when broodstock fish will have to be maintained in captivity for a very long term. The overall annual increase of spine erosion in captivity is likely to be underestimated because all the captive fish spines were sampled only in summer, when the *SS/TS* ratio is physiologically higher. In some instances, the erosion process in the oldest age class specimens was prominent and resulted in spine fracture during sectioning (authors’ personal observation).

The present data on spine bone resorption fit into the wider context of systemic dysfunctions occurring in ABFT experimentally or commercially reared in captivity. The reproductive axis appeared to be seriously affected by the rearing conditions: luteinizing hormone was insufficiently released from pituitary gonadotrophs [22], gonads did not grow properly [12, 14], oocytes failed to mature and went into atresia [12, 13], proliferation of male germ cells was reduced and apoptosis increased [14, 23], and 11-ketotestosterone plasma concentrations were lower than physiological levels [14]. Reproductive activity of reared ABFT is further and dramatically impaired by an acute exposure to stress, with 100% vitellogenic oocytes undergoing atresia 24 h after experiencing a single stressing event [16]. Moreover, juvenile ABFT reared in captivity in the Adriatic Sea had a high density of liver melanomacrophage centers [35] and an increased tumor necrosis factor gene expression [34], associated to hepatocyte anti cytochrome P4501A immunopositivity [35] and apoptosis [34], indicating a remarkable susceptibility to environmental stress.

Altogether, the above mentioned literature data, along with the present results, demonstrate a severe fragility of this species to the various stressors associated with confinement in captivity. As for other fish species reared in captivity, different potential causes have been hypothesized as the cause of the reproductive dysfunctions reported for the ABFT [12–14], such as captivity-induced stress; the lack of the appropriate “natural” spawning environment; or even a lack of essential components in the diet.

These same factors may also affect bone metabolism and spine resorption. The role of sex steroids, 17β estradiol (E_**2**_) in particular, in fish bone metabolism has not yet been fully demonstrated. In general E_**2**_, which in mammals is a potent inhibitor of bone resorption, has the opposite effect in fish. However, in rainbow trout (*Onchoriunchus mykiss*), E_**2**_ induces calcium mobilization from scales and decreases skeleton bone resorption and osteoclastic activity [56, 57]. Although it was never demonstrated, it is postulated that, due to an inadequate pituitary gonadotropin release, E_2_ plasma levels remain low in captive-reared ABFT which can affect bone metabolism.

Although the physiological role of vitamin D3 (1,25-dihydroxy vitamin D) in fish remains to be clarified, injection of vitamin D3 into mature female European eels resulted in a stimulation of bone formation and a reduction of osteoclastic resorption [58]. Captive-reared ABFT are fed only frozen fish and this may decrease vitamin D3 availability and consequently decrease bone deposition and increase osteoclastic resorption.

Cortisol is another potential factor responsible for spine bone resorption in ABFT reared in captivity. This hormone is a powerful stimulator of bone demineralization in fish [59] and its plasma levels might increase in stressed captive ABFT [16].

Although dorsal fins have active roles in fish swimming mechanics—they come into action during braking [60] interacting with the other median fins (anal and caudal) as well as during propulsion and maneuvering [61–63]—the role of spiny rays is still to be fully understood [64]. Whatever their function, the first dorsal spine has to resist hydrodynamic loading during swimming, which is likely lower in confined situations. since ABFT are usually reared in round sea cages 50 to 90 m in diameter, where they swim slowly and synchronously in a circular uniform motion, unless external stimuli intervene (e.g. food supply or cage maintenance operations). Fish bone undergoes a continuous modeling which is related to the mechanical strains that the skeleton element is exposed to [65] so the limited use of the dorsal fins during the fish’s circular motion in rearing cages might unbalance the bone resorption/deposition process in favor of material loss.

## Conclusions

This paper described the trend of the bone resorption process of the first spine of the first dorsal fin of wild ABFT during the first 13 years of life. The physiological, progressive spine bone loss occurring in wild individuals dramatically increases in captive conditions. The hypothetical cause or causes of this phenomenon might be related to nutritional deficiencies, alteration of the endocrine profile, cortisol-induced stress, or loss of spine functions during locomotion in rearing conditions. The bone loss reported in the present paper represents an additional aspect to be added to all other systemic changes already described. Further research is required in order to clarify and remove the causes of all these dysfunctions in order to improve the well-being of this species in captivity.

## Supporting Information

S1 Table Total, solid bone tissue and reabsorbed bone tissue surfaces of first dorsal spine section in *Thunnus thynnus*. Data from 186 wild and 242 captive-reared Mediterranean specimens grouped according their age.
(XLSX)
